# Assessment of Medication Adherence Among Patients With Hypertension and Diabetes Mellitus in a Tertiary Healthcare Center: A Descriptive Study

**DOI:** 10.7759/cureus.63126

**Published:** 2024-06-25

**Authors:** Rahul Govindani, Amiya Sharma, Narmada Patel, Pooja Baradia, Abhimanyu Agrawal

**Affiliations:** 1 Department of General Medicine, LN Medical College and Research Center, Bhopal, IND; 2 Department of Internal Medicine, Mahaveer Institute of Medical Sciences and Research, Bhopal, IND

**Keywords:** diabetes mellitus, morisky medication adherence scale-8 (mmas-8), drug compliance, hypertension, diabetes self-management questionnaire, medication adherence strategies

## Abstract

Introduction

Effective management of diabetes and hypertension requires a comprehensive approach, including dietary adjustments, physical activity, routine screening for complications, and adherence to medication. Proper adherence to pharmacotherapy is essential for maintaining glycemic control and managing blood pressure. Non-adherence can result in increased complications, higher healthcare costs, and greater morbidity and mortality. This study aimed to evaluate the sociodemographic profile and medication adherence among adults with diabetes and hypertension, focusing on those undergoing treatment. The objective was to determine the extent of adherence and identify factors that influence adherence among patients with type 2 diabetes mellitus (T2DM) and hypertension.

Methods

A community-based study was conducted on patients with T2DM and/or hypertension in both rural and urban areas of Bhopal, specifically those attending JK Hospital, Bhopal, Madhya Pradesh, India. Participants were selected using a simple random sampling method. Medication adherence was assessed using the eight-item Morisky Medication Adherence Scale (MMAS-8), a validated tool for measuring patient adherence behavior.

Results

The study included 300 participants, of whom 68% (n=204) were male and 32% (n=96) were female. The average age of the participants was 57.52±10.3 years. Among the 300 patients, 9% (n=27) had high adherence to medication, 24.7% (n=74) had moderate adherence, and 66.3% (n=199) had low adherence.

Conclusions

Effective strategies must include targeted patient education on medication costs, side effects, and the long-term benefits of adherence. Integrating technological aids like smartphone apps may enhance adherence. Patients who adhere closely to their regimens are more likely to achieve better control over blood pressure and glycated hemoglobin (HbA1c) levels, underscoring the critical importance of adherence in managing chronic conditions effectively.

## Introduction

Diabetes mellitus is a group of metabolic disorders where glucose is underutilized as an energy source and overproduced due to inappropriate gluconeogenesis and glycogenolysis, resulting in hyperglycemia [1]. Systemic arterial hypertension (hereafter referred to as hypertension) is characterized by persistently high blood pressure (BP) in the systemic arteries. BP is commonly expressed as the ratio of systolic BP (that is, the pressure that the blood exerts on the arterial walls when the heart contracts) and diastolic BP (the pressure when the heart relaxes) [2,3].

Both diabetes mellitus and hypertension rank among the most prevalent diseases and cardiovascular risk factors globally, with their occurrence rising as individuals age. Elevated BP is frequently observed in patients with type 2 diabetes mellitus (T2DM), potentially stemming from the influence of insulin resistance on vascular and renal functions. Conversely, growing evidence indicates a higher prevalence of carbohydrate metabolism disorders in hypertensive individuals, suggesting a bidirectional pathogenic relationship between diabetes mellitus and hypertension [4-6].

India has the second-highest number of diabetic patients in the world. In the age group of 20-79 years, there were 74.9 million diabetics in India in 2021 [7]. This number is projected to increase to 124.9 million by 2045. The International Diabetes Federation (IDF) reports that one out of every eight diabetic adults globally resides in India, and one in every three households in India has a diabetic patient. This highlights the significant and growing impact of diabetes on the country's population and healthcare system [7]. The IDF predicts that global healthcare expenditures related to diabetes will surge to $1.03 trillion by 2030 and $1.05 trillion by 2045. Diabetes, along with hypertension and dyslipidemia, stands out as major risk factors for cardiovascular disease (CVD) [7].

In India, the prevalence of hypertension is 24-30% and 12-14% in urban and rural areas, respectively [8]. Worldwide, 13.5% of premature deaths, 54% of strokes, and 47% of ischemic heart disease cases were attributed to hypertension [9]. An estimated 1.56 billion adults will be suffering from high BP by 2025 [8].

The WHO divides the various factors that influence treatment adherence into five categories: socioeconomics, healthcare team and service delivery system, disease, treatment, and patient-related factors [10]. Although some of these factors cannot be changed, patient-related factors can be modified through training and increasing patients' awareness and knowledge. However, a change in awareness and knowledge does not always result in a change in attitude, and a change in attitude does not always result in a change in behavior because the individual's environment may not allow them to act in a certain way [10].

Assessing medication adherence and its determinants among patients with non-communicable diseases (NCDs) can inform targeted interventions. Non-adherence to medication is associated with increased rates of hospitalization, mortality, and out-of-pocket expenses among NCD patients [11]. Suboptimal adherence to established care standards significantly contributes to the development of complications in NCDs. Therefore, medication adherence is crucial for achieving optimal therapeutic outcomes in the management of diabetes and hypertension [12].

Objectives

The objective of the study was to evaluate medication adherence among diabetic patients at LN Medical College & JK Hospital, Bhopal, Madhya Pradesh, India, and to assess the sociodemographic characteristics of patients with diabetes and hypertension.

## Materials and methods

A cross-sectional study was conducted over three months from January 2023 to March 2023 at the tertiary healthcare center, LN Medical College & JK Hospital. Inclusion criteria were patients with diabetes and/or hypertension with a duration of disease of more than one year and willing to provide consent. Pregnant or lactating individuals and those unwilling to provide consent were excluded. The study was approved by the Institutional Ethics Committee of LN Medical College and Research Centre & JK Hospital (approval number: LNMC & RC/Dean/2022/Ethics/040).

Sample size

A sample size of 256 patients was taken as per the crude prevalence of diabetes and hypertension in India was 7.5% (95%CI, 7.3-7.7%) and 25.3% (95% CI, 25.0-25.6%), respectively [13]. All the patients were selected by using the simple random sampling method. A total of 400 participants had diabetes and/or hypertension with a duration of disease of more than one year. Of these, 300 patients fulfilled the inclusion/exclusion criteria and were finally included in the study.

Data collection

Data collection involved interviews using a pre-tested, semi-structured questionnaire along with the eight-item Morisky Medication Adherence Scale (MMAS-8) (see Appendix) [14,15]. The MMAS-8 has been validated for use in several countries with several groups of patients and has been found to be a valid and reliable scale to measure adherence in patients with hypertension, diabetes, osteoporosis, epilepsy, myocardial infarction, and patients taking warfarin [12,16]. The MMAS-8 is a self-reporting questionnaire designed to measure medication adherence. It consists of eight questions that assess behaviors related to medication-taking. The first seven questions are yes/no questions that address specific patterns of non-adherence, such as forgetting to take medication or stopping medication when feeling better or worse. The eighth question uses a 5-point Likert scale to gauge the frequency of taking medication over a specified period.

Patients were categorized based on their adherence levels as high, moderate, or low adherence according to their MMAS-8 scores. Total scores on the MMAS-8 range from 0 to 8, with scores of 8 reflecting high adherence, 7 or 6 reflecting medium adherence, and <6 reflecting low adherence [14,15].

When using the MMAS-8 to assess medication adherence in patients with diabetes and hypertension, several confounding factors can affect results. These include the complexity of the medication regimen, presence of comorbidities, cognitive function, socioeconomic status, health literacy, support systems, medication side effects, mental health conditions, healthcare access, and cultural beliefs. Addressing these factors is essential for accurate assessment and improving adherence in these patient populations.

Definitions

The American Diabetes Association (ADA) defines diabetes using several criteria: an A1C level of 6.5% or higher, fasting plasma glucose (FPG) of 126 mg/dL or higher, two-hour plasma glucose (2-h PG) of 200 mg/dL or higher during an oral glucose tolerance test (OGTT), or a random plasma glucose of 200 mg/dL or higher with symptoms of hyperglycemia. In the absence of unequivocal hyperglycemia, diagnosis requires two abnormal test results from the same or different samples [17]. The BP thresholds that define hypertension depend on the measurement method. The 2023 European Society of Hypertension (ESH) guidelines retain the same BP classifications as the 2018 European Society of Cardiology (ESC)/ESH guidelines. According to the ESH, hypertension is diagnosed at a threshold of >140/90 mm Hg (grade 1), whereas the American College of Cardiology (ACC)/American Heart Association (AHA) guidelines use a lower threshold of >130/80 mm Hg (stage 1). The ACC/AHA guidelines categorize systolic BP of 130-139 mm Hg or diastolic BP of 80-89 mm Hg as stage 1 hypertension, while the ESH guidelines consider these ranges as "normal" or "high-normal" (130-139/85-89 mm Hg). The ACC/AHA defines BP ≥140/90 mm Hg as stage 2 hypertension, whereas the ESH subdivides BP ≥140/90 mm Hg into grades 1, 2, and 3 hypertension [18]. Medication adherence, as defined by the International Society for Pharmacoeconomics and Outcomes Research, is "the extent to which a patient acts in accordance with the prescribed interval and dose of a dosing regimen” [19].

Statistical analysis

Data was compiled using Microsoft Excel (Microsoft Corporation, Redmond, Washington, United States) and analyzed with IBM SPSS Statistics for Windows, Version 22.0 (Released 2013; IBM Corp., Armonk, New York, United States). Descriptive statistics for all quantitative and qualitative data were presented as frequencies and percentages. In the statistical analysis, we used chi-square tests of independence to examine the association between medication adherence levels (low, moderate, and high) with various sociodemographic characteristics (age, gender, education, occupation, and socioeconomic status) and treatment profile (disease, duration, and number of pills) among patients with hypertension and T2DM. 

## Results

The study, conducted over a three-month period and involving 300 patients, provided detailed insights into the demographics and socioeconomic status of the participants. These socio-demographic characteristics are summarized in Table 1. The largest age group was between 51 and 70 years, with a mean age of 57.52 years and a standard deviation of 10.3 years. A significant portion of the participants had limited formal education: 33% had no education, and 26% had only primary education, indicating potential challenges in health literacy within this group. Employment data revealed that 21% of the participants were unemployed, and a further 38.34% were engaged in unskilled labor, suggesting a considerable portion of the sample was economically vulnerable. Socioeconomic classification using the modified Kuppuswamy scale (2023) indicated that 67% of the subjects belonged to the lower and upper-lower classes, emphasizing the economic challenges faced by a majority of the patients. This socio-economic backdrop provides an important context for understanding the health and lifestyle issues faced by the study population.

**Table 1:** Sociodemographic characterstics of the study population (N=300)

Variables	Number	Percentage
Age		
18-50 years	69	23%
51-70 years	189	63%
>70 years	42	14%
Gender		
Male	204	68%
Female	96	32%
Education		
No Education	99	33%
Primary school	78	26%
Secondary school	60	20%
Higher secondary school	36	12%
Graduation	27	9%
Marital status		
Unmarried	28	9.34%
Married	224	74.66%
Separated	45	15%
Widowed	3	1%
Occupation		
Unemployed	63	21%
Unskilled	115	38.34%
Semiskilled	47	15.66%
Skilled	33	11%
Self business	29	9.66%
Professional	13	4.34%
Socioeconomic status		
Lower	92	30.66%
Upper lower	109	36.34%
Lower middle	47	15.66%
Upper middle	36	12%
Upper class	16	5.34%
Religion		
Hindu	260	86.66%
Muslim	40	13.34%

The study provided a comprehensive analysis of clinical characteristics, particularly focusing on the prevalence of chronic diseases and their management. Among the patients, diabetes was the most common condition, affecting 217 individuals (72.34%). Within this diabetic subgroup, 143 patients had diabetes mellitus alone, while 74 had both diabetes and another comorbid condition, indicating a significant burden of diabetes in this population.

A deeper look into the duration of disease revealed that 50.66% of the study population had been living with their condition for more than five years. This long-term disease group included 34 individuals with hypertension alone, 68 with diabetes mellitus alone, and 50 with both hypertension and diabetes. Despite the extended duration of their primary conditions, other significant comorbidities were relatively less common. Specifically, coronary artery disease, stroke, chronic kidney disease, dyslipidemia, and chronic inflammatory conditions were present in only 29% of the total population. 

The study also examined medication regimens, revealing a preference for dual medication therapy. A total of 146 participants (48.66%) were on a dual medication regimen, which was more common than single medication therapy or regimens involving three or more medications. This indicates that combination drug therapy is a widely adopted and potentially more effective strategy for managing the chronic conditions prevalent in this patient population.

The comprehensive clinical and treatment characteristics of the study participants are shown in Table 2

**Table 2:** Comprehensive clinical and treatment characteristics of study participants (N=300)

Characteristics	Number	Percentage
Disease		
Hypertension	83	27.66%
Diabetes Mellitus	143	47.66%
Both	74	24.68%
Duration of hypertension (N=83)		
<5 years	49	59%
>5 years	34	41%
Duration of diabetes (N=143)		
<5 years	75	52.5%
>5 years	68	47.5%
Duration of diabetes and hypertension (N=74)		
<5 years	24	32.5%
>5 years	50	67.5%
Other co-morbidities (coronary artery disease, stroke, chronic kidney disease, dyslipidemia, and chronic inflammatory conditions)		
Present	87	29%
Absent	213	71%
Number of medications		
One	72	24%
Two	146	48.66%
Three	61	20.34%
Four and more	21	7%

**Figure 1:**
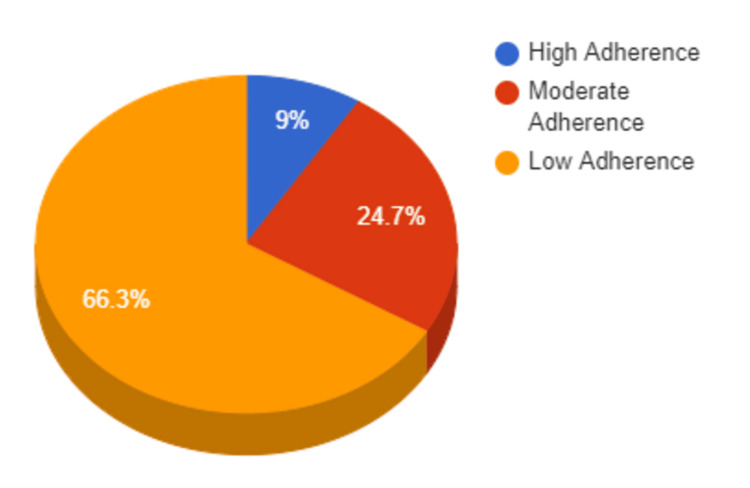
Level of medication adherence among the study population (N=300)

High, moderate, and low adherence to medication was found among 27 (9%), 74 (24.7%), and 199 (66.3%) participants, respectively (Figure 1).

The study found that a higher proportion of individuals over 50 years old, from lower socioeconomic classes, male, unemployed, and unskilled, had low adherence. Conversely, those with good education levels exhibited high medication adherence (Table 3).

**Table 3:** Distribution of sociodemographic profile with adherence level among study population Chi-square tests of independence were used to calculate the p-values; p-values indicate statistically significant associations between medication adherence levels and socio-demographic characteristics (p < 0.05).

Characterstics	Low Adherence (N=199)	Moderate Adherence (N=74)	High Adherence (N=27)	Total (N=300)	p value
Age					
18-50 years	27(13.6%)	26(35.2%)	16(59.2%)	69	p value <0.0001
51-70 years	145(72.8%)	34(45.9%)	10(37%)	189	
>70 years	27(13.6%)	14(18.9%)	1(3.8%)	42	
Gender					
Male	155(77.9%)	39(52.7%)	10(37%)	204	p value <0.0001
Female	44(22.1%)	35(47.3%)	17(63%)	96	
Education					
No Education	95(47.3%)	4(5.4%)	0	99	p value <0.0001
Primary school	67(33.7%)	10(13.5%)	1(3.7%)	78	
Secondary school	24(12%)	30(40.5%)	6(22.2%)	60	
Higher secondary school	10(5%)	18(24.3%)	8(29.6%)	36	
Graduation	2(2%)	13(16.3%)	12(44.5%)	27	
Occupation					
Unemployed	59(29.6%)	4(5.4%)	0	63	p value <0.0001
Unskilled	90(45.2%)	25(33.8%)	0	115	
Semiskilled	31(15.6%)	15(20.3%)	1(3.8%)	47	
Skilled	14(7.1%)	13(17.5%)	6(22.2%)	33	
Self business	4(2%)	15(20.3%)	10(37%)	29	
Professional	1(0.5%)	2(2.7%)	10(37%)	13	
Socioeconomic status					
Lower	86(43.2%)	4(5.4%)	2(7.5%)	92	p value <0.0001
Upper lower	74(37.2%)	30(40.5%)	5(18.5%)	109	
Lower middle	32(16.1%)	8(10.8%)	7(25.9%)	47	
Upper middle	5(2.5%)	25(33.8%)	6(22.2%)	36	
Upper class	2(1%)	7(9.5%)	7(25.9%)	16	

A low level of adherence was observed among patients with diabetes and both conditions, accounting for 156 (78.4%) participants. As the disease duration increased, the adherence level decreased, with 122 (61.35%) exhibiting low adherence. Similarly, patients on two or more medications also showed a low level of adherence, at 76.38%. This information is detailed in Table 4.

**Table 4:** Distribution of treatment profile with adherence level Chi-square tests of independence were used to calculate the p-values; p-values indicate statistically significant associations between medication adherence levels and treatment profile (p < 0.05).

Characteristics	Low Adherence (N=199)	Moderate Adherence (N=74)	High Adherence (N=27)	Total (N=300), n	p-value
Disease, n (%)					
Hypertension	43 (21.6%)	24 (32.4%)	16 (59.25%)	83	0.000046
Diabetes Mellitus	94 (47.25%)	40 (54.1%)	9 (33.34%)	143	
Both	62 (31.15%)	10 (13.5%)	2 (7.41%)	74	
Duration of disease, n					
<5 years	77	50	21	148	<0.0001
>5 years	122	24	6	152	
Number of medications, n (%)					
One	24 (12.11%)	28 (37.9%)	20 (74.1%)	72	<0.0001
Two	110 (55.27%)	32 (43.2%)	4 (14.8%)	146	
Three	47 (23.62%)	12 (16.2%)	2 (7.4%)	61	
Four and more	18 (9%)	2 (2.7%)	1 (3.7%)	21	

Other factors like alternative medication (n=121; 40.33%), missed doses due to a busy schedule (n=112; 37.33%), drug cost (n=57; 19%), and side effects (n=9; 3%) were reasons for non-adherence (Table 5).

**Table 5:** Reasons for non-adherence among the study population (N=300)

Reason for non-adherence	Number	Percentage
Discontinued due to alternative medication	121	40.33%
Forgot due to busy schedule	112	37.33%
Drug cost	57	19%
Side effect	9	3%

## Discussion

The majority of participants in this study (n=189; 63%) were within the 51-70 age group, with a mean age of 57.52 years. This finding highlights a critical period during which conditions like hypertension and diabetes often become clinically apparent, despite potentially being asymptomatic for many years prior. Many studies overlook the age at which a condition first manifests, focusing instead on the age at which it is detected. This is significant because conditions like hypertension and diabetes can remain undiagnosed and untreated for extended periods, leading to complications by the time they are identified.

In a study by Shukla et al., the mean age of participants with diabetes was 57.52±12.33 years, reflecting similar trends [20]. Likewise, a study conducted in the urban slums of Hyderabad found that the average age of individuals diagnosed with hypertension was 54.5±11.03 years [21]. These findings collectively suggest that middle to late adulthood is a critical window for the manifestation and subsequent detection of these chronic conditions. This underscores the importance of early screening and preventive measures in this age group. By identifying and managing these health issues before they become symptomatic and cause significant health problems, healthcare providers can improve outcomes and reduce the burden of chronic diseases. Early intervention strategies, regular health check-ups, and increased awareness about these conditions in people approaching middle age can help in mitigating long-term health risks.

Being health-conscious is crucial for preventing diseases, yet the educational background of the participants in this study was notably low. A significant 59% had no education or only completed primary school, and only 21% had completed higher education. This low level of education can impact health literacy, which is essential for understanding and adhering to medical advice and treatment plans. Similarly, in a study conducted in the urban slums of Hyderabad, the majority (64.9%) of individuals were found to be illiterate [21]. This lack of education can hinder an individual's ability to access, understand, and use health information, which is critical for disease prevention and management.

The socioeconomic status of the participants further compounds this issue. A large proportion (67%) of the study participants were from lower and upper-lower socioeconomic backgrounds. Similarly, an investigation conducted in Mumbai found that a significant majority (50.5%) of participants belonged to the upper-lower socioeconomic class [22]. Socioeconomic status is well-documented to play a crucial role in determining adherence to medical treatment and overall health outcomes. Individuals from lower socioeconomic backgrounds often face multiple barriers to accessing healthcare, including financial constraints, limited access to healthcare facilities, and lack of health insurance. They may also prioritize immediate economic needs over long-term health investments. Furthermore, lower socioeconomic status is often associated with higher levels of stress, poor living conditions, and inadequate nutrition, all of which can exacerbate health problems.

The combined effects of low education and socioeconomic status create significant obstacles to maintaining good health. These factors can lead to poor health behaviors, delayed healthcare seeking, and lower adherence to prescribed treatments. For example, individuals with limited education may not understand the importance of taking medications regularly or attending follow-up appointments. They might also be less aware of preventive measures, such as routine screenings and healthy lifestyle choices.

Therefore, addressing these educational and socioeconomic disparities is crucial for improving health outcomes. Efforts should be made to enhance health education, improve access to healthcare services, and provide support for those from lower socioeconomic backgrounds. This could include community health programs, educational campaigns, and policies aimed at reducing financial barriers to healthcare. By tackling these issues, it is possible to promote better health behaviors, improve disease prevention, and ensure more effective management of chronic conditions.

The study observed a decline in medication adherence as the duration of illness increased, with 61.35% of patients showing low adherence when the disease had persisted for more than five years. Similarly, a study conducted in Patiala found that only 13.79% of patients diagnosed with diabetes within the past one to five years exhibited high adherence [20]. Long-term management of chronic conditions can lead to patient fatigue and decreased motivation, highlighting the need for strategies to sustain adherence over time.

In this study, high, moderate, and low adherence to medication was found among 27 (9%), 74 (24.7%), and 199 (66.3%) study subjects, respectively. This highlights a significant challenge in achieving high adherence rates, particularly in managing chronic conditions. Comparatively, a study by Shukla et al. reported that 52% of patients had low adherence, 29% had moderate adherence, and 19% had high adherence according to the MMAS-8 item scores [20]. Arulmozhi et al. in Puducherry found that 39% of patients exhibited low adherence using the same scale [23]. Meanwhile, a community-based cross-sectional study in a rural village in Kerala revealed that 74% of diabetic patients were less adherent to their medication regimen [24].

Our study found a higher proportion of high adherence (59.25%) among patients aged less than 50 years. This is consistent with findings by Shaimol et al., who observed that medication adherence decreased with increasing age [25]. However, in a study by Thekkur et al., low adherence was observed in only 12.8% of participants above 60 years, suggesting variability in adherence across different populations and age groups [26].

A study conducted in Hyderabad found that 61.7% of participants demonstrated high adherence to their antihypertensive medications. Similarly, Rao et al. found that 60.6% of participants were adherent to their prescribed medication [27]. Bhandari et al. reported a treatment adherence rate of 73% (95%CI, 68-78%), indicating a relatively high adherence in their study population [28]. In a coastal population in South India, compliance rates were notably high, with 82.2% for hypertension treatment and 83.6% for T2DM [29]. Contrastingly, the study by Thomas et al. in Bengaluru reported that only 50.3% of participants adhered to their antihypertensive medication regimen [30]. These findings underscore the complexity of medication adherence across different demographics and conditions.

In this study, only 21% of participants were prescribed a single medication a day. This finding is in contrast with a study conducted in Puducherry, where a slightly different picture emerged: the majority of patients (40.6%) were consuming their medication only once a day [26]. This discrepancy suggests variability in prescribing practices and patient adherence across different regions.

The number of medications a patient is required to take is particularly significant when it comes to medication adherence. In this study, it was observed that 74.1% of patients who were prescribed a single medication showed high adherence to their treatment regimen. In stark contrast, only 25.9% of patients who were required to take two or more medications per day exhibited the same level of adherence. This highlights the burden that a complex medication regimen can place on patients, making it more challenging for them to follow their prescribed treatment. Complex regimens often involve multiple doses at different times of the day, which can be difficult for patients to remember and manage, especially for those with busy schedules or cognitive impairments. Additionally, taking multiple medications can increase the risk of missed doses, incorrect dosing, and medication errors, further reducing the effectiveness of the treatment. Simplified medication regimens, such as once-daily dosing, have been shown to improve adherence rates [31]. When patients are required to take fewer medications, it is easier for them to integrate their medication routine into their daily lives, thereby increasing the likelihood of consistent adherence. This is particularly important for chronic conditions, where long-term adherence is critical for managing the disease and preventing complications.

The findings from this study underscore the need for healthcare providers to consider the complexity of medication regimens when developing treatment plans. By prioritizing simplified regimens, healthcare providers can help improve patient adherence, leading to better health outcomes. This may involve prescribing combination medications, where multiple medications are combined into a single medication, or choosing medications that require less frequent dosing.

Limitations of the study

Limitations include reliance on self-reported data, potentially introducing recall and social desirability biases. The cross-sectional design limits establishing causality or long-term trends. The sample size of 300 may lack generalizability, and the study's single-center setting may limit broader applicability. Exclusion criteria like pregnancy may introduce selection bias.

## Conclusions

These findings underscore the multifaceted nature of adherence challenges, influenced by factors such as high medication burdens, long-term management requirements, economic constraints, and the presence of multiple chronic conditions. Effective strategies to improve adherence must include targeted patient education and counseling sessions that address medication costs, potential side effects, and the long-term benefits of adherence in managing chronic diseases and preventing complications. Healthcare providers should also consider integrating technological solutions like smartphone reminder apps and automated medication dispensers, which have shown promise in enhancing adherence by facilitating regular medication schedules and improving medication-taking behaviors.

Furthermore, initiatives aimed at improving health literacy and promoting self-management skills among patients, particularly those with limited education, are crucial. By addressing these barriers comprehensively and tailoring interventions to the specific needs of at-risk patient populations, healthcare systems can strive to achieve better adherence rates and ultimately improve health outcomes for individuals managing chronic conditions like hypertension and diabetes.

## Appendices

**Table 6:** MMAS-8 questionnaire MMAS-8: eight-item Morisky Medication Adherence Scale Reference: [14,15]; Used with a license agreement from Donald E. Morisky, Department of Community Health Sciences, UCLA School of Public Health, Los Angeles, California

Questions
Do you sometimes forget to take your prescribed medicines?
Over the past 2 weeks, were there any days when you did not take your prescribed medicines?
Have you stopped taking medications because you feel worse when you took it?
When you travel or leave home, do you sometimes forget to bring along your medications?
Did you take your prescribed medicine yesterday?
When you feel like your health is under control, do you sometime stop taking your medications?
Do you feel hassled about sticking to your prescribed treatment plan?
How often do you have difficulty remembering to take all your prescribed medicines?
